# Genome-wide identification of methylated CpG sites in nongenital cutaneous warts

**DOI:** 10.1186/s12920-020-00745-6

**Published:** 2020-07-08

**Authors:** Laith N. AL-Eitan, Mansour A. Alghamdi, Amneh H. Tarkhan, Firas A. Al-Qarqaz

**Affiliations:** 1 Department of Applied Biological Sciences, Jordan University of Science and Technology, Irbid, 22110 Jordan; 2 Department of Biotechnology and Genetic Engineering, Jordan University of Science and Technology, Irbid, 22110 Jordan; 3 Department of Anatomy, College of Medicine, King Khalid University, Abha, 61421 Saudi Arabia; 4 Genomics and Personalized Medicine Unit, College of Medicine, King Khalid University, Abha, 61421 Saudi Arabia; 5 Department of Internal Medicine, Jordan University of Science and Technology, Irbid, 22110 Jordan; 6 Division of Dermatology, Department of Internal Medicine, King Abdullah University Hospital Jordan University of Science and Technology, Irbid, 22110 Jordan

**Keywords:** HPV, Warts, DNA methylation, CpG, Epigenetics

## Abstract

**Background:**

Low-risk HPV infection has not been the subject of epigenetic investigation. The present study was carried out in order to investigate the methylation status of CpG sites in non-genital cutaneous warts.

**Methods:**

Genomic DNA was extracted from 24 paired epidermal samples of warts and normal skin. DNA samples were bisulfite converted and underwent genome-wide methylation profiling using the Infinium MethylationEPIC BeadChip Kit.

**Results:**

From a total of 844,234 CpG sites, 56,960 and 43,040 CpG sites were found to be hypo- and hypermethylated, respectively, in non-genital cutaneous warts. The most differentially methylated CpG sites in warts were located within the *C10orf26*, *FAM83H-AS1*, *ZNF644*, *LINC00702*, *GSAP*, *STAT5A*, *HDAC4*, *NCALD*, and *EXOC4* genes.

**Conclusion:**

Non-genital cutaneous warts exhibit a unique CpG methylation signature.

## Background

CpG sites are parts of DNA that consist of a cytosine nucleotide linked to a guanine nucleotide by a phosphate group, and they are often found as a part of CpG islands, the latter of which are areas of high CpG frequencies [1]. From an epigenetic perspective, CpGs are of particular importance due to the fact that DNA methylation in mammals occurs primarily in a CpG context [2]. In mammalian genomes, the majority of CpG sites are methylated, while those in CpG islands are generally hypomethylated [3]. Due to the high mutability of methylcytosine, methylated CpG sites are under-represented in the human genome [4]. Aberrant CpG methylation patterns increase susceptibility to various diseases, including cancer, but such changes can also be induced during host-pathogen interactions [5, 6].

Host gene dysregulation is a common component of viral infection, and such changes are often generated via epigenetic exploitation of the host genome [7]. In order to evade the antiviral immune response, DNA viruses induce aberrant methylation of immune-related genes in the host [8]. One such example is the human papillomavirus (HPV), a DNA virus that alters host methylation patterns as a part of its life cycle and replication mechanisms within keratinocytes [9]. To date, more than 200 HPV genotypes have been characterized, most of which are low-risk and often manifest in the form of benign cutaneous or genital lesions known as warts [10]. However, a small group of HPV types are considered to be high risk, as they are a causative agent for several different types of squamous cell carcinomas [11].

High-risk HPV infection affects cervical cancer progression by increasing levels of DNA methylation, although methylation patterns were heterogenous among different neoplastic grades [12–14]. Hypomethylation of a CpG site in the *MAL* gene was reported to be potentially associated with persistent cervical infection with high-risk HPV [15]. Moreover, HPV-positive head-and-neck squamous cell carcinomas exhibited a novel methylation signature in which hypomethylated CpG islands were functionally correlated with gene expression [16]. In fact, HPV-induced epigenetic changes are a major contributing factor to the stability of malignant head-and-neck squamous cell carcinoma [17]. Similarly, CpG loci were differentially methylated in HPV-positive anal squamous neoplasia, and significant differential methylation was observed between in-situ and invasive samples [18].

Unlike its high-risk counterpart, low-risk HPV infection has not been the subject of epigenetic analysis in the context of non-genital cutaneous warts, the latter of which constitutes an extremely common skin disease that is benign and self-limiting in the majority of cases [19]. The most prevalent type of non-genital cutaneous wart is the common wart, which usually manifests on the hands and feet as a firm, hyperkeratotic papule with an irregular surface [20]. The extensive transformation that an HPV-infected keratinocyte undergoes to form a wart suggests that a similar change in methylation patterns must occur. Subsequently, the aim of the current study is to identify the genome-wide methylation status of CpG sites in warts as compared to normal skin.

## Methods

### Patient recruitment

Twelve patients were recruited at the dermatological clinic in King Abdullah University Hospital in the north of Jordan. The Institutional Review Board (IRB) at Jordan University of Science and Technology (JUST) granted ethical approval to conduct the present study. The inclusion criteria for participants comprised the following characteristics: being male, being free from autoimmune disease, presenting with common warts, not having received prior treatment for their warts, and having given written informed consent. Shave biopsies were performed by a resident dermatologist in order to excise paired normal skin and wart samples from each patient, which were then stored at − 20 °C until subsequent processing.

### Extraction of genomic DNA and bisulfite conversion

RNA-free genomic DNA was extracted by means of the QIAamp DNA Mini Kit (Qiagen, Germany) and shipped to the Australian Genome Research Facility (AGRF) on dry ice. Upon arriving to the AGRF, further quality control analysis was performed for each sample using the QuantiFluor® dsDNA System (Promega, USA) and 0.8% agarose gel electrophoresis to determine their purity and integrity, respectively. After obtaining assurance of their quality, the EZ DNA Methylation kit (Zymo Research, USA) was employed for the bisulfite conversion of normalized samples.

### Genome-wide methylation profiling and data processing

The Infinium MethylationEPIC BeadChip Kit (Illumina, USA) was utilized in order to interrogate over 850,000 methylation sites. The MethylationEPIC array contains 866,895 probes that target 863,904 CpG sites, 2932 CpH sites, and 59 rs sites. The raw intensity data generated by the array was analyzed using RnBeads, a computational R package [21].

### Differential methylation analysis

To calculate the extent of differential methylation (DM) for each CpG site, limma was used to determine three ranks: the beta difference in methylation means between warts (W) and normal skin (NS), the log_2_ of the quotient in methylation, and the DM *p*-value [21]. Limma was also utilized to compute *p*-values on CpG sites [22]. Multiple testing was corrected for by setting the false discovery rate (FDR) at 5% with the Benjamini-Hochberg procedure. Using these three ranks, a combined rank was formulated in which increased DM at a particular CpG site resulted in a smaller rank [21]. The combined rank was used to sort DM CpG sites in ascending order, and the top-ranking 100,000 sites were selected for further analysis.

### Enrichment, pathway, and signaling analysis

Gene ontology (GO) term enrichment analysis as well as KEGG and Reactome pathway analysis of the top 100 CpG sites were carried out using the Database for Annotation, Visualization, and Integrated Discovery (DAVID) v6.8 (https://david.ncifcrf.gov/). GO terms revolved around three criteria (biological process (BP), cellular component (CC), and molecular function (MF)), and the cut-off threshold was fixed at *p*-value ≤0.05. After selecting the top-ranked 100 DM CpG sites, the Signaling Network Open Resource 2.0 (SIGNOR) was used to analyze the signaling networks of associated genes [23].

## Results

### Sample clustering

**Fig. 1:**
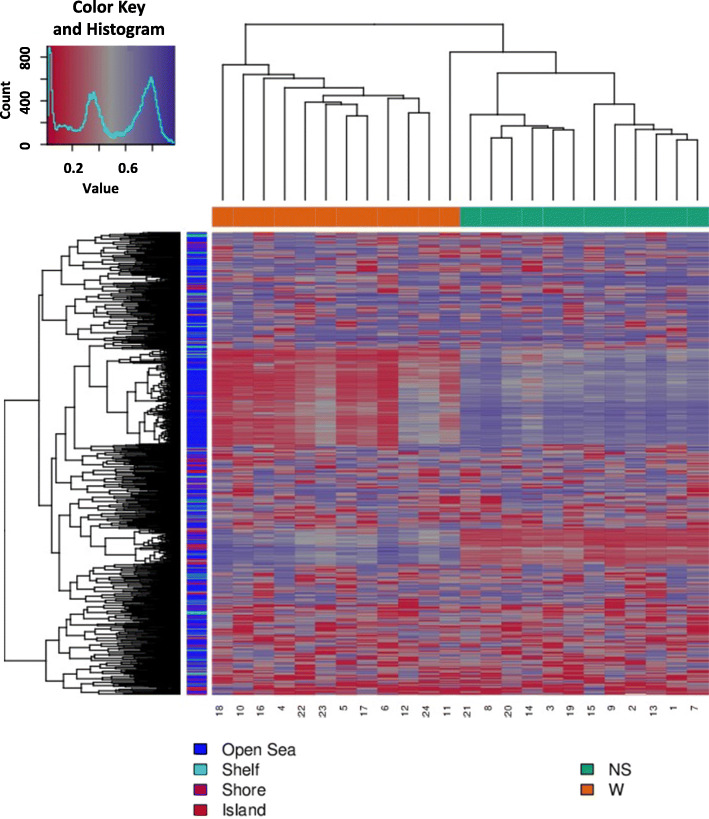
Heatmap showing the hierarchal clustering of the top 1000 most variable loci across all 24 samples. Clustering used average linkage and Manhattan distance. Patient identification numbers are shown on the x-axis. W and NS stand for wart and normal skin, respectively

**Fig. 2:**
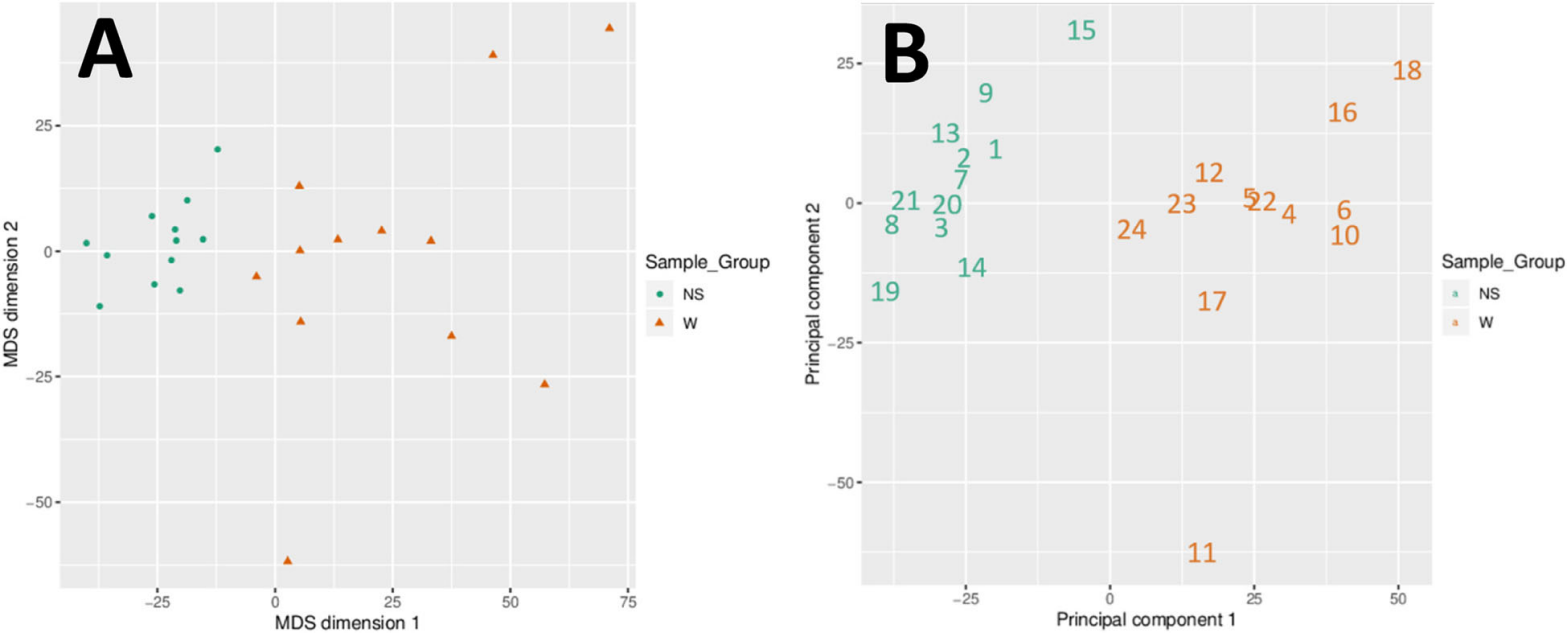
Scatter plots showing the coordinates of the wart (W) and normal skin (NS) samples (**a**) after performing Kruskal’s multi-dimensional scaling based on the matrix of the average methylation levels and Euclidean distance and (**b**) on the first and second principal components. A clear difference between the W and NS samples can be seen in both plots

Based on the DM values of the top-ranking 1000 loci, an expected clustering pattern can be observed between the NS and W samples (Fig. 1). Using multidimensional scaling (MDS) and principal component analysis (PCA), strong signals in sample methylation values were examined (Fig. 2a and b).

### Processing and filtering of data

**Fig. 3:**
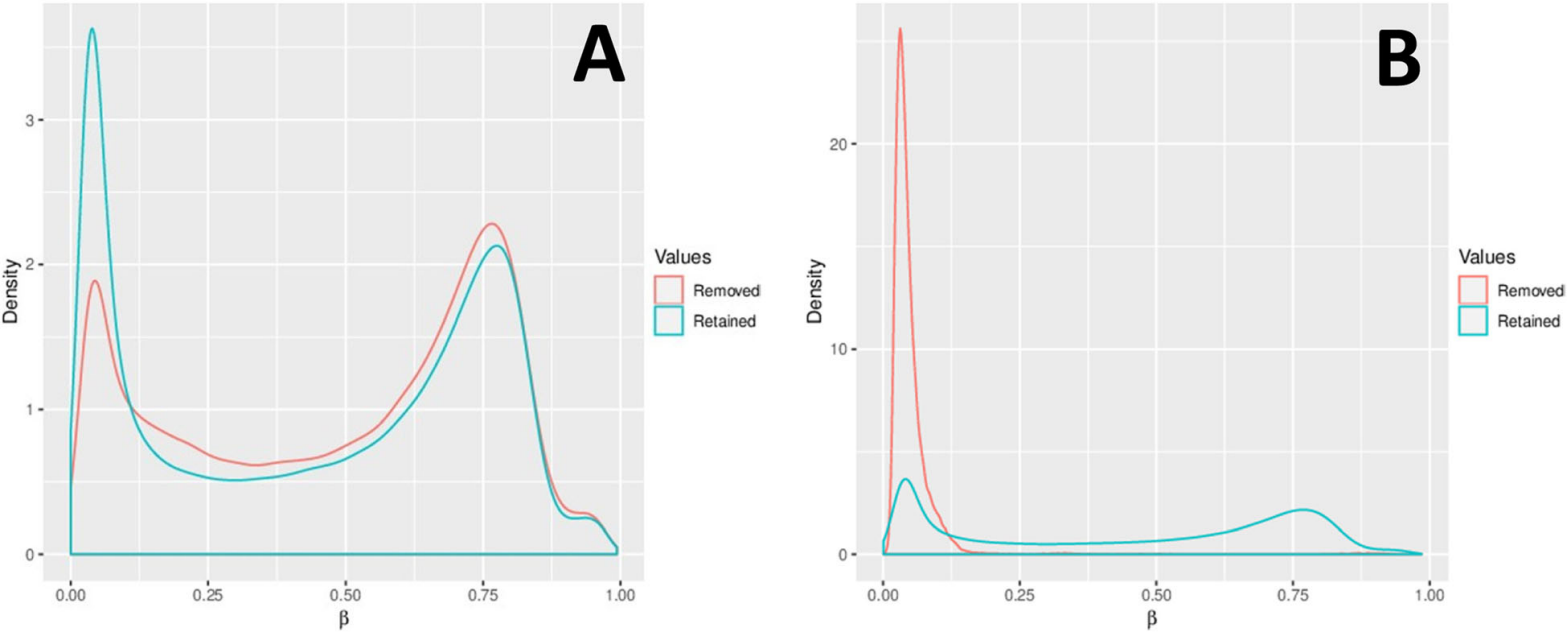
Contrasting the density distributions of methylation levels (β) after (**a**) removal of SNP-enriched probes and filtration by Greedycut and (**b**) removal of context-specific probes

**Fig. 4:**
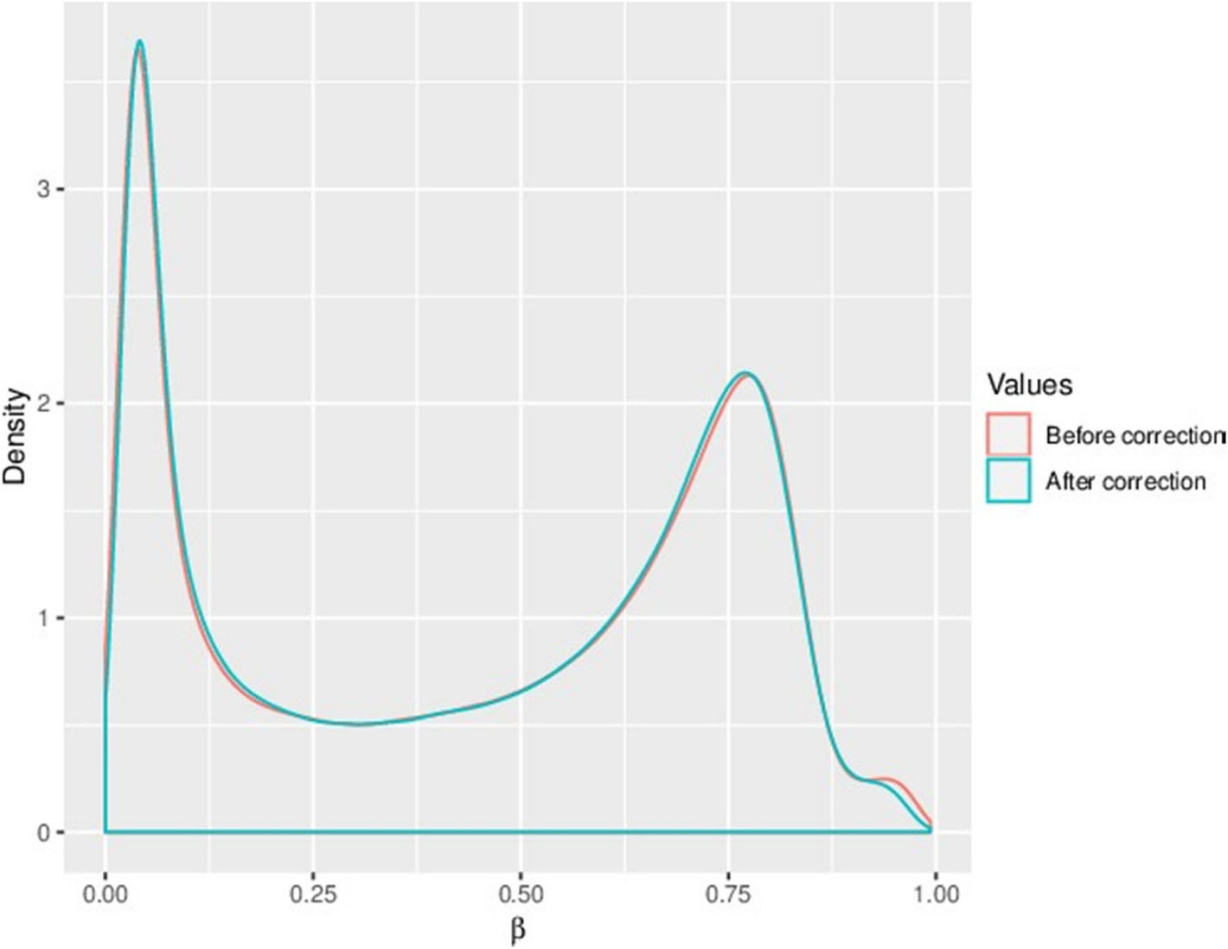
Density distributions of methylation levels (β) were normalized using Dasen’s method. The figure compares the β values before and after correction

17,371 probes were removed due to their overlap with SNPs (Fig. 3a). A further 2,310 probes were filtered out using the Greedycut algorithm in RnBeads. Additional filtering eliminated 2,980 probes with specific contexts (Fig. 3b). In total, 22,661 probes were removed and 844,234 probes were retained. Both probes and samples were subject to the full RnBeads package pipeline, which entailed quality control, preprocessing, batch effects testing, and normalization (Fig. 4). The complete processed methylation data for the CpG sites can be found in Supplementary File.

### Differential methylation of CpG sites

**Fig. 5:**
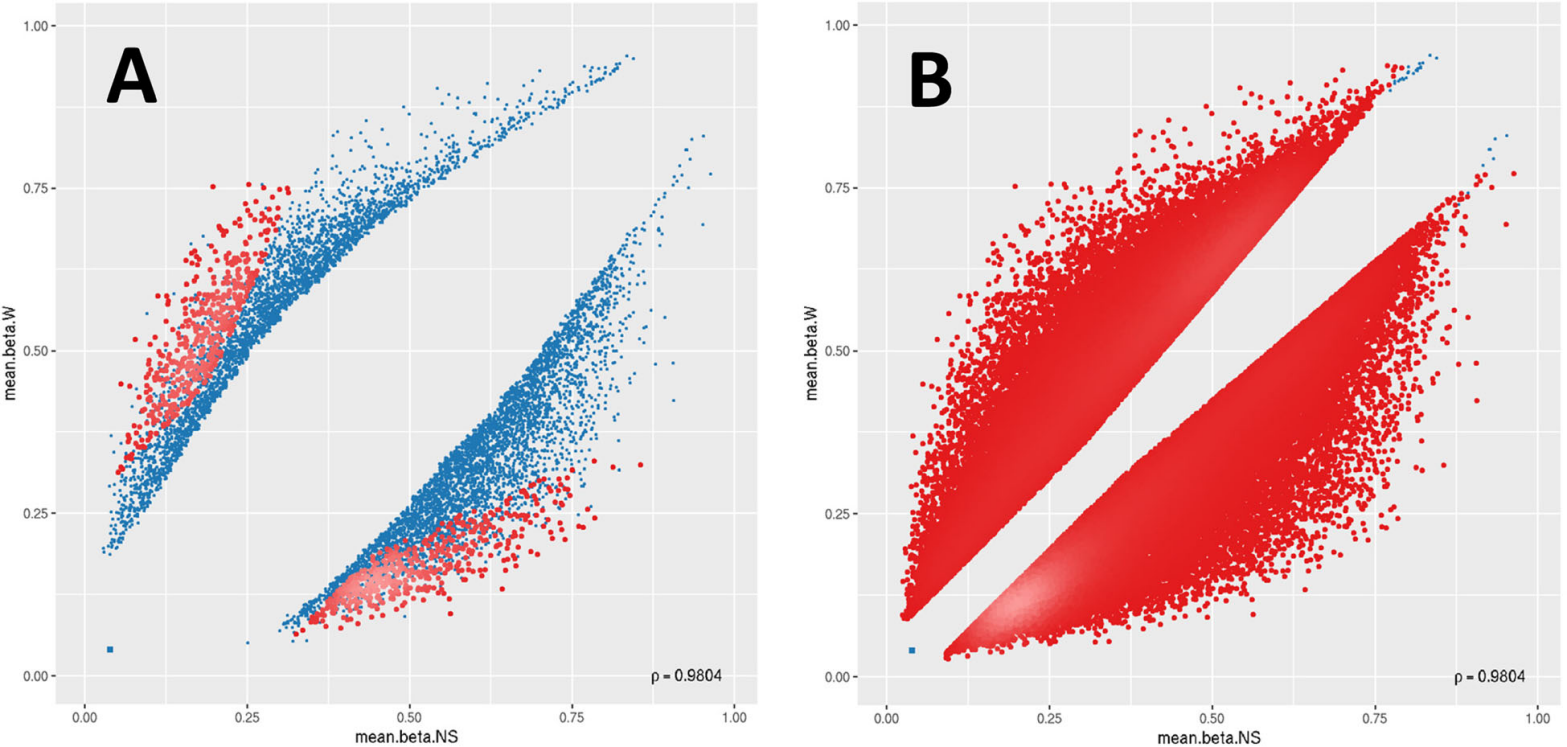
Scatter plots for the (**a**) top-ranking 1000 and (**b**) top-ranking 100,000 differentially methylated CpG sites. For each plot, the mean β values of normal skin (mean.beta. NS) are on the x-axis, while the mean β values of warts (mean.beta. W) are on the y-axis. Methylation levels (β) varied between 0 (unmethylated) and 1 (fully methylated). Blue points represent variable differentially methylated sites

**Fig. 6:**
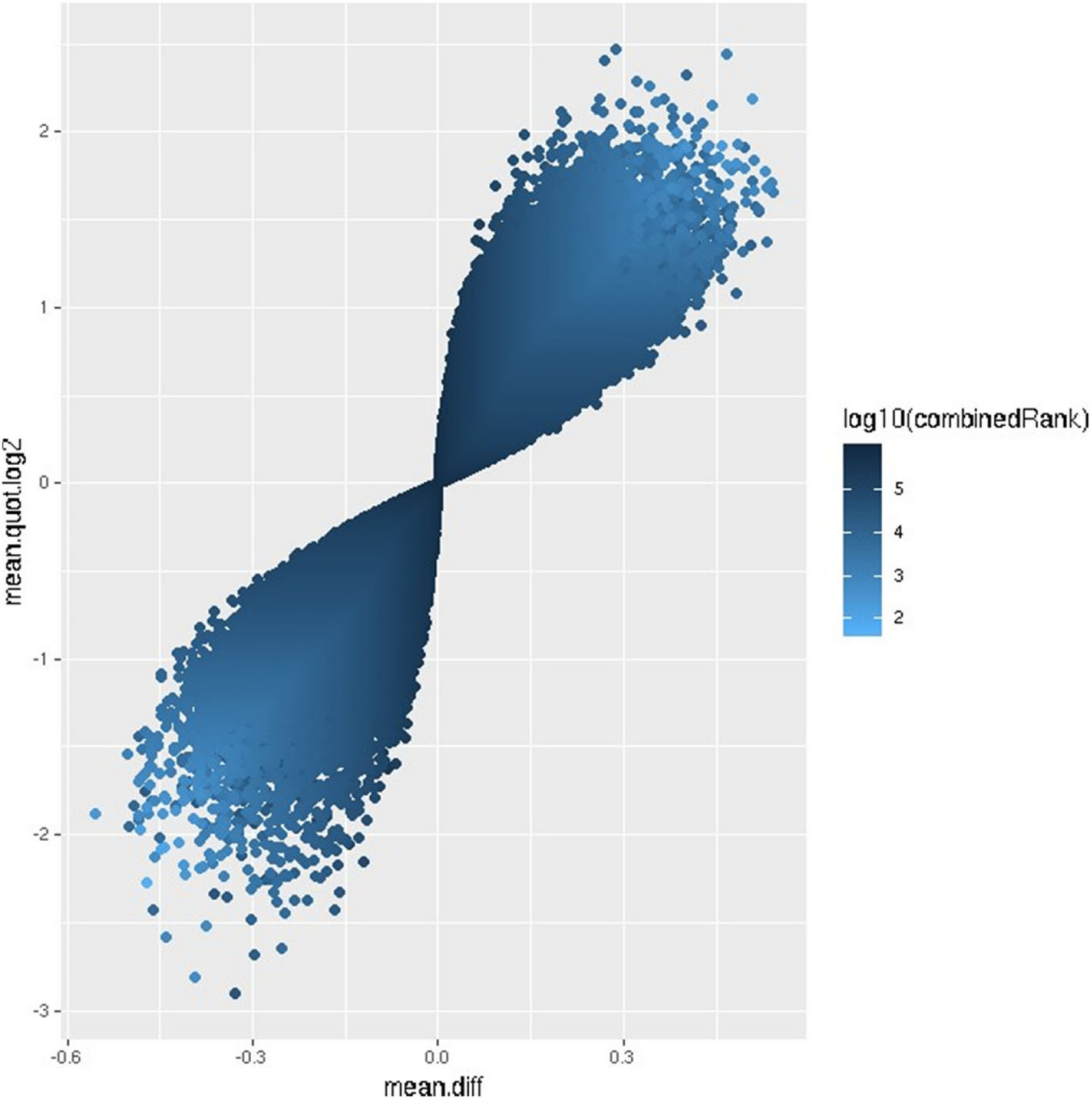
Volcano plot of the top-ranking 1000 differentially methylated sites. Differential methylation was measured by the log2 of the mean quotient in methylation (mean.quot.log2) and the mean fold difference (mean.diff) between warts (W) and normal skin (NS). Data points less than 0 represent relative hypomethylation, while those more than 0 represent relative hypermethylation. The intensity of each data point correlates with the combined rank score as shown on the color scale to the right

**Fig. 7:**
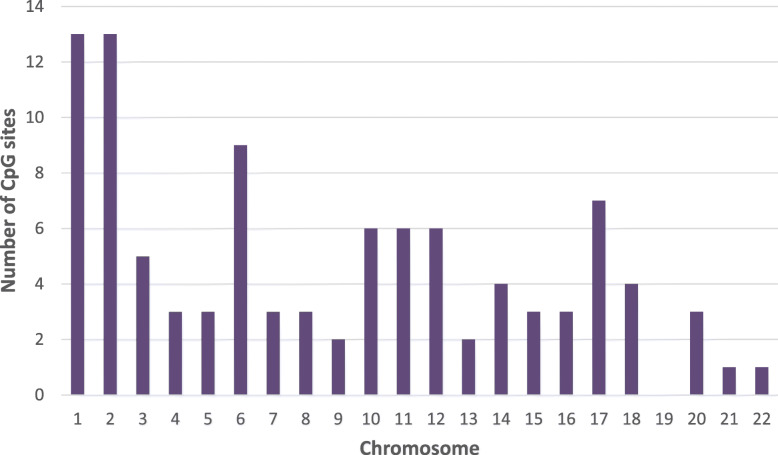
Chromosomal distribution of the top 100 differentially methylated CpG sites in warts compared to normal skin

Of the top-ranking 100,000 CpG sites in terms of DM, 56,960 sites were hypomethylated and 43,040 sites were hypermethylated in W compared to NS, with a mean beta difference greater than 0.055 and less than − 0.055 (*p*-value < 0.032; adjusted p-value < 0.032) (Fig. 5). The beta difference for the hypomethylated and hypermethylated sites ranged from − 0.055 to 0.56 and 0.55 to 0.56, respectively. Similarly, the log_2_ of the quotient in methylation between W and NS ranged from − 2.47 to 2.9 (Fig. 6). The highest concentration of DM sites was seen on chromosomes 1 and 2 (Fig. 7). The top-ranking100 CpG sites, i.e. the most DM, are listed in Table 1.

**Table 1:** The 100 CpG sites with the lowest combined rank scores

CpG	Chromosome	Gene	Methylation region	CpG Island	Mean β value (NS)	Mean β value (W)	Mean β value diff (W-NS)	mean.quot. (log2)	P-value	False discovery rate	Combined rank score	Methylation pattern
cg09671951	10	C10orf26	Body		0.1129	0.5848	0.4719	2.2753	6.82E-16	5.09E-11	48	Hypermethylation
cg27071672	8	FAM83H- AS1	Body	S_Shelf	0.1290	0.5765	0.4475	2.0772	1.74E-14	1.99E-10	102	Hypermethylation
cg07385604	1	ZNF644	TSS1500	S_Shore	0.1281	0.5720	0.4440	2.0756	9.33E-16	5.09E-11	110	Hypermethylation
cg12432168	10	LINC00702	Body		0.1558	0.6389	0.4832	1.9690	6.83E-15	1.31E-10	151	Hypermethylation
cg06305962	7	GSAP	Body		0.1249	0.5457	0.4208	2.0421	1.49E-14	1.91E-10	183	Hypermethylation
cg00071017	2				0.6112	0.1537	−0.4575	−1.9241	7.83E-16	5.09E-11	186	Hypomethylation
cg16530881	17				0.1080	0.5208	0.4127	2.1688	1.99E-13	7.07E-10	236	Hypermethylation
cg08246644	17	STAT5A	TSS1500;5’UTR;TS S200	N_Shore	0.1009	0.5098	0.4089	2.2286	2.29E-15	7.97E-11	245	Hypermethylation
cg05171197	2	HDAC4	Body		0.1973	0.7523	0.5550	1.8785	1.65E-13	6.32E-10	247	Hypermethylation
cg16516970	8	NCALD	5’UTR		0.1567	0.6028	0.4461	1.8783	2.74E-14	2.4E-10	248	Hypermethylation
cg03432603	7	EXOC4	Body		0.6423	0.1335	−0.5088	−2.1842	2.14E-13	7.24E-10	249	Hypomethylation
cg01890417	1	ZNF644	TSS1500	S_Shore	0.1519	0.5773	0.4254	1.8592	2.75E-14	2.4E-10	274	Hypermethylation
cg00194325	2	TANC1	Body		0.1719	0.6446	0.4727	1.8473	5.54E-16	5.09E-11	290	Hypermethylation
cg25894955	9	ABCA1	Body		0.5371	0.1351	−0.4021	−1.9151	2.81E-14	2.4E-10	295	Hypomethylation
cg10560060	13	GJB2	5’UTR	N_Shelf	0.6623	0.1799	−0.4824	− 1.8238	2.4E-13	7.66E-10	329	Hypomethylation
cg10144055	2				0.1350	0.5324	0.3974	1.9032	7.42E-15	1.34E-10	336	Hypermethylation
cg19342952	13	GJB2	5’UTR	N_Shore	0.6449	0.1770	−0.4679	−1.8080	5.82E-14	3.81E-10	347	Hypomethylation
cg15612257	2			N_Shore	0.1547	0.5655	0.4108	1.8048	2.56E-15	7.97E-11	359	Hypermethylation
cg07863022	17	SEPT9;	5’UTR;Body;TSS15 00		0.1681	0.6076	0.4395	1.7937	3.99E-15	9.85E-11	375	Hypermethylation
cg02745009	3	ARHGAP3 1	Body	S_Shore	0.1718	0.6135	0.4417	1.7783	2.9E-13	8.24E-10	407	Hypermethylation
cg15782771	5				0.7396	0.2096	−0.5299	−1.7709	3.84E-14	3.03E-10	428	Hypomethylation
cg04272613	14	DAAM1	5’UTR		0.1508	0.5378	0.3869	1.7680	2.74E-15	7.97E-11	445	Hypermethylation
cg10017626	2			N_Shore	0.0988	0.4854	0.3866	2.1870	2.02E-13	7.07E-10	449	Hypermethylation
cg18248499	11	ROBO4	TSS1500		0.5057	0.1193	−0.3865	−1.9961	3.43E-13	9.24E-10	451	Hypomethylation
cg10841463	14				0.1646	0.5798	0.4153	1.7566	7.01E-17	1.69E-11	457	Hypermethylation
cg19497037	11				0.5188	0.1328	−0.3860	−1.8891	7.48E-13	1.37E-09	459	Hypomethylation
cg13800897	2				0.5754	0.1613	−0.4141	−1.7727	8.99E-13	1.55E-09	490	Hypomethylation
cg13632752	8				0.5831	0.1474	−0.4357	−1.9140	9.15E-13	1.56E-09	494	Hypomethylation
cg27277339	15	MYO5C	Body		0.1561	0.5455	0.3894	1.7417	9.65E-14	4.88E-10	496	Hypermethylation
cg19158326	22	GRAMD4	Body		0.0980	0.4793	0.3813	2.1796	3.91E-15	9.85E-11	514	Hypermethylation
cg20400915	17	STAT5A	TSS1500;5’UTR;TS S200	N_Shore	0.0555	0.4492	0.3937	2.8086	1.02E-12	1.66E-09	519	Hypermethylation
cg20392201	1	FAM129A	Body		0.1263	0.5848	0.4585	2.1258	1.04E-12	1.69E-09	521	Hypermethylation
cg21879102	12	CIT	Body	N_Shore	0.1946	0.6605	0.4659	1.7127	2.61E-13	7.91E-10	549	Hypermethylation
cg14384093	9	C9orf5	Body	N_Shelf	0.1256	0.5097	0.3841	1.9381	1.25E-12	1.9E-09	557	Hypermethylation
cg18813270	2	HS1BP3- IT1	TSS1500		0.6868	0.1911	−0.4957	−1.7929	1.3E-12	1.95E-09	564	Hypomethylation
cg19449565	2	HDAC4	Body		0.1691	0.6536	0.4845	1.8898	1.33E-12	1.96E-09	570	Hypermethylation
cg09187774	10				0.6165	0.1627	−0.4538	−1.8593	1.34E-12	1.98E-09	572	Hypomethylation
cg07980148	4			S_Shelf	0.6475	0.1624	−0.4852	−1.9317	1.36E-12	1.99E-09	573	Hypomethylation
cg03304533	11				0.6668	0.1977	−0.4690	−1.7040	3.09E-13	8.67E-10	576	Hypomethylation
cg08569613	17	STAT5A	TSS1500;5’UTR;TS S200	N_Shore	0.0692	0.4453	0.3761	2.5226	6.22E-15	1.25E-10	578	Hypermethylation
cg06848849	1	ARHGEF10 L	Body		0.1451	0.5204	0.3753	1.7737	2.84E-14	2.4E-10	591	Hypermethylation
cg17164954	6	ARID1B	Body	S_Shelf	0.1656	0.5604	0.3948	1.6997	6.39E-13	1.24E-09	591	Hypermethylation
cg13733684	15	ZNF106	TSS200;Body		0.1724	0.5807	0.4083	1.6954	1.72E-14	1.99E-10	603	Hypermethylation
cg05669832	2	PRKD3	TSS1500		0.2068	0.6911	0.4843	1.6934	2.72E-13	8.02E-10	611	Hypermethylation
cg06382539	12	BHLHE41	Body	N_Shore	0.1759	0.5882	0.4123	1.6864	1.58E-12	2.14E-09	629	Hypermethylation
cg16303737	20				0.5411	0.1618	−0.3793	−1.6819	7.37E-13	1.36E-09	642	Hypomethylation
cg27335585	5	LOC101929 710	Body		0.7606	0.2298	−0.5308	− 1.6840	1.78E-12	2.31E-09	652	Hypomethylation
cg09185727	6				0.5467	0.1642	−0.3825	−1.6763	2.73E-13	8.02E-10	652	Hypomethylation
cg15350314	3	LOC101928 992	Body		0.1552	0.5574	0.4021	1.7797	1.85E-12	2.36E-09	658	Hypermethylation
cg11508674	14	FOXN3	Body		0.1648	0.6344	0.4696	1.8820	2.02E-12	2.49E-09	683	Hypermethylation
cg06610988	18	SETBP1	5’UTR	S_Shore	0.1684	0.5546	0.3862	1.6622	3.94E-14	3.06E-10	684	Hypermethylation
cg18492160	15				0.5276	0.1311	−0.3965	−1.9299	2.03E-12	2.49E-09	690	Hypomethylation
cg02921273	20				0.0980	0.4645	0.3664	2.1349	3.95E-14	3.06E-10	699	Hypermethylation
cg14167109	11	MAML2	Body		0.1594	0.5381	0.3787	1.6939	2.13E-12	2.55E-09	703	Hypermethylation
cg06373653	12	CD163L1	Body		0.4932	0.1277	−0.3656	−1.8699	2.3E-13	7.47E-10	709	Hypomethylation
cg09403144	18	SETBP1	Body		0.1549	0.5202	0.3653	1.6847	3.68E-14	2.96E-10	714	Hypermethylation
cg06746371	6	DCBLD1	Body		0.7344	0.2249	−0.5095	−1.6641	2.31E-12	2.68E-09	727	Hypomethylation
cg14002969	20	PTPRA	5’UTR		0.4985	0.1342	−0.3644	−1.8187	5.04E-13	1.11E-09	727	Hypomethylation
cg07076915	16	PKD1	Body	N_Shelf	0.2112	0.6851	0.4739	1.6517	2.48E-14	2.32E-10	728	Hypermethylation
cg27341747	6				0.2010	0.6524	0.4514	1.6503	5.21E-14	3.61E-10	732	Hypermethylation
cg20964957	4				0.5612	0.1185	−0.4428	−2.1527	2.39E-12	2.74E-09	736	Hypomethylation
cg19917507	18	ALPK2	Body		0.5863	0.1813	−0.4050	−1.6401	4.78E-13	1.08E-09	757	Hypomethylation
cg00925616	1			Island	0.0781	0.5172	0.4392	2.5818	2.61E-12	2.89E-09	762	Hypermethylation
cg13515269	12	BHLHE41	3’UTR	N_Shore	0.2078	0.6886	0.4809	1.6818	2.71E-12	2.96E-09	772	Hypermethylation
cg18638180	21	C21orf70	Body	S_Shore	0.1734	0.6318	0.4584	1.8073	2.93E-12	3.13E-09	791	Hypermethylation
cg17967134	17	MPRIP	Body		0.1283	0.4884	0.3601	1.8495	1.19E-12	1.83E-09	804	Hypermethylation
cg06373648	6	SYNGAP1	Body		0.1564	0.5160	0.3596	1.6604	4.57E-13	1.06E-09	818	Hypermethylation
cg14825152	1				0.1422	0.5010	0.3588	1.7475	4.83E-13	1.09E-09	828	Hypermethylation
cg08966889	6	TRAM2	Body	N_Shore	0.1747	0.5588	0.3840	1.6224	1.16E-12	1.81E-09	828	Hypermethylation
cg09443467	5	TENM2	Body		0.5807	0.1623	−0.4185	−1.7779	3.44E-12	3.49E-09	833	Hypomethylation
cg17758398	18				0.6251	0.1850	−0.4401	−1.7035	3.48E-12	3.51E-09	836	Hypomethylation
cg01821452	12				0.2138	0.6779	0.4641	1.6198	1.44E-12	2.06E-09	840	Hypermethylation
cg19663114	3	MED12L	Body		0.7670	0.2279	−0.5390	−1.7073	3.64E-12	3.6E-09	853	Hypomethylation
cg10624729	1	FAM73A	Body		0.1847	0.5864	0.4017	1.6152	1.53E-13	6.05E-10	857	Hypermethylation
cg26586287	11				0.6087	0.1625	−0.4463	−1.8430	3.74E-12	3.67E-09	859	Hypomethylation
cg23983887	1	VPS13D	Body		0.1546	0.5113	0.3567	1.6629	1.65E-12	2.21E-09	866	Hypermethylation
cg08921063	6	WASF1	5’UTR		0.4750	0.1185	−0.3565	−1.9164	2.02E-12	2.49E-09	871	Hypomethylation
cg14359656	17	SPAG9	Body		0.5856	0.1477	−0.4380	−1.9176	3.98E-12	3.81E-09	883	Hypomethylation
cg26754187	3				0.5241	0.1368	−0.3873	−1.8634	4E-12	3.81E-09	885	Hypomethylation
cg10126884	4				0.4827	0.1254	−0.3573	−1.8635	4.05E-12	3.85E-09	888	Hypomethylation
cg13355857	16				0.6967	0.1872	−0.5096	−1.8418	4.06E-12	3.85E-09	889	Hypomethylation
cg13568540	7	PKD1L1	Body		0.6599	0.1847	−0.4752	−1.7828	4.22E-12	3.95E-09	901	Hypomethylation
cg08611640	1	VPS13D	Body;Body		0.1109	0.4654	0.3546	1.9757	7.7E-15	1.34E-10	912	Hypermethylation
cg25322618	2	RAPGEF4	TSS200;Body		0.2041	0.6388	0.4347	1.5994	1.22E-13	5.62E-10	913	Hypermethylation
cg16669099	6				0.1801	0.5652	0.3851	1.5971	3.77E-12	3.69E-09	919	Hypermethylation
cg19712663	6	SLC22A23	Body		0.1017	0.4711	0.3694	2.1069	4.47E-12	4.07E-09	927	Hypermethylation
cg13720639	14	SIPA1L1	Body		0.1299	0.4946	0.3646	1.8502	4.5E-12	4.08E-09	929	Hypermethylation
cg04394003	12	C12orf75	TSS1500	N_Shore	0.1172	0.4703	0.3531	1.9170	3.46E-12	3.51E-09	931	Hypermethylation
cg17356718	2	HDAC4	Body		0.1435	0.5270	0.3835	1.8066	4.51E-12	4.08E-09	931	Hypermethylation
cg26639076	2	RIF1	3’UTR		0.1710	0.5360	0.3650	1.5930	7.11E-14	4.2E-10	936	Hypermethylation
cg07969739	10	BTAF1	Body		0.5137	0.1346	−0.3791	−1.8564	4.74E-12	4.17E-09	958	Hypomethylation
cg26125625	3	SLC12A8	Body	Island	0.1074	0.4587	0.3513	1.9968	2.24E-12	2.64E-09	965	Hypermethylation
cg18251218	1				0.0952	0.4461	0.3510	2.1169	1.17E-16	1.69E-11	967	Hypermethylation
cg23909079	10	GRID1	Body		0.6723	0.2146	−0.4577	−1.6031	4.92E-12	4.25E-09	977	Hypomethylation
cg24117274	1	RAP1GAP	Body	N_Shelf	0.1260	0.4766	0.3505	1.8387	7.37E-14	4.29E-10	979	Hypermethylation
cg09262171	16	ADCY9	Body		0.1896	0.5865	0.3970	1.5796	3.41E-14	2.78E-10	992	Hypermethylation
cg14600452	10				0.6088	0.1865	−0.4223	−1.6550	5.44E-12	4.53E-09	1014	Hypomethylation
cg24088496	11	MAML2	Body		0.1856	0.5727	0.3871	1.5747	1.73E-13	6.44E-10	1016	Hypermethylation
cg06968781	1	GMEB1	5’UTR		0.5323	0.1666	−0.3657	−1.6189	5.65E-12	4.63E-09	1030	Hypomethylation
cg03133881	1	MAST2	Body		0.5066	0.1589	−0.3477	−1.6128	5.41E-12	4.52E-09	1035	Hypomethylation

### Functional enrichment analysis

GO enrichment analyses of the genes associated with the top 100 DM CpG sites were performed using the DAVID webtool. Table 2 shows the most significant GO terms (*p*-value ≤0.05). Associated genes were mainly enriched for “SH3 domain binding”, “actin binding”, and “GTPase activator activity” on the MF level, “regulation of GTPase activity” and “positive regulation of GTPase” on the BP level, and “postsynaptic membrane” on the CC level. The most significant KEGG and Reactome pathway terms with a *p*-value ≤0.05 are presented. The genes were mainly enriched in the Rap1 signaling and VxPx cargo-targeting to cilium pathways (Table 3).

**Table 2:** GO enrichment analyses revealed significant (p-value ≤0.05) GO terms and associated enriched genes in the biological process (BP), cellular component (CC), and molecular function (MF) categories

Category	Term	P-value	Genes
MF	GO:0017124 ~ SH3 domain binding	0.004	ARHGAP31, ZNF106, SYNGAP1, CIT
MF	GO:0003779 ~ actin binding	0.006	NCALD, WASF1, DAAM1, MPRIP, MYO5C
MF	GO:0005096 ~ GTPase activator activity	0.006	ARHGAP31, RAP1GAP, SIPA1L1, SYNGAP1, ARHGEF10L
BP	GO:0043087 ~ regulation of GTPase activity	0.014	RAP1GAP, SIPA1L1, SYNGAP1
BP	GO:0043547 ~ positive regulation of GTPase activity	0.019	ARHGAP31, RAP1GAP, PTPRA, RAPGEF4, SYNGAP1, ARHGEF10L
CC	GO:0045211 ~ postsynaptic membrane	0.019	SIPA1L1, TENM2, TANC1, GRID1
BP	GO:0016337 ~ single organismal cell-cell adhesion	0.031	TENM2, PKD1, PKD1L1
BP	GO:0050982 ~ detection of mechanical stimulus	0.038	PKD1, PKD1L1
MF	GO:0017016 ~ Ras GTPase binding	0.039	RAP1GAP, RAPGEF4
BP	GO:0010832 ~ negative regulation of myotube differentiation	0.043	HDAC4, BHLHE41
BP	GO:0018105 ~ peptidyl-serine phosphorylation	0.046	MAST2, PKD1, PRKD3

**Table 3:** The most significantly enriched KEGG and Reactome pathway terms of the genes associated with the top-ranking 100 DM CpG sites

Category	Term	P-value	Genes
KEGG_PATHWAY	hsa04015:Rap1 signaling pathway	0.001	RAP1GAP, ADCY9, SIPA1L1, RAPGEF4, PRKD3
REACTOME_PATHWAY	R-HSA-5620916:VxPx cargo-targeting to cilium	0.045	EXOC4, PKD1

### Signaling network analysis

**Fig. 8:**
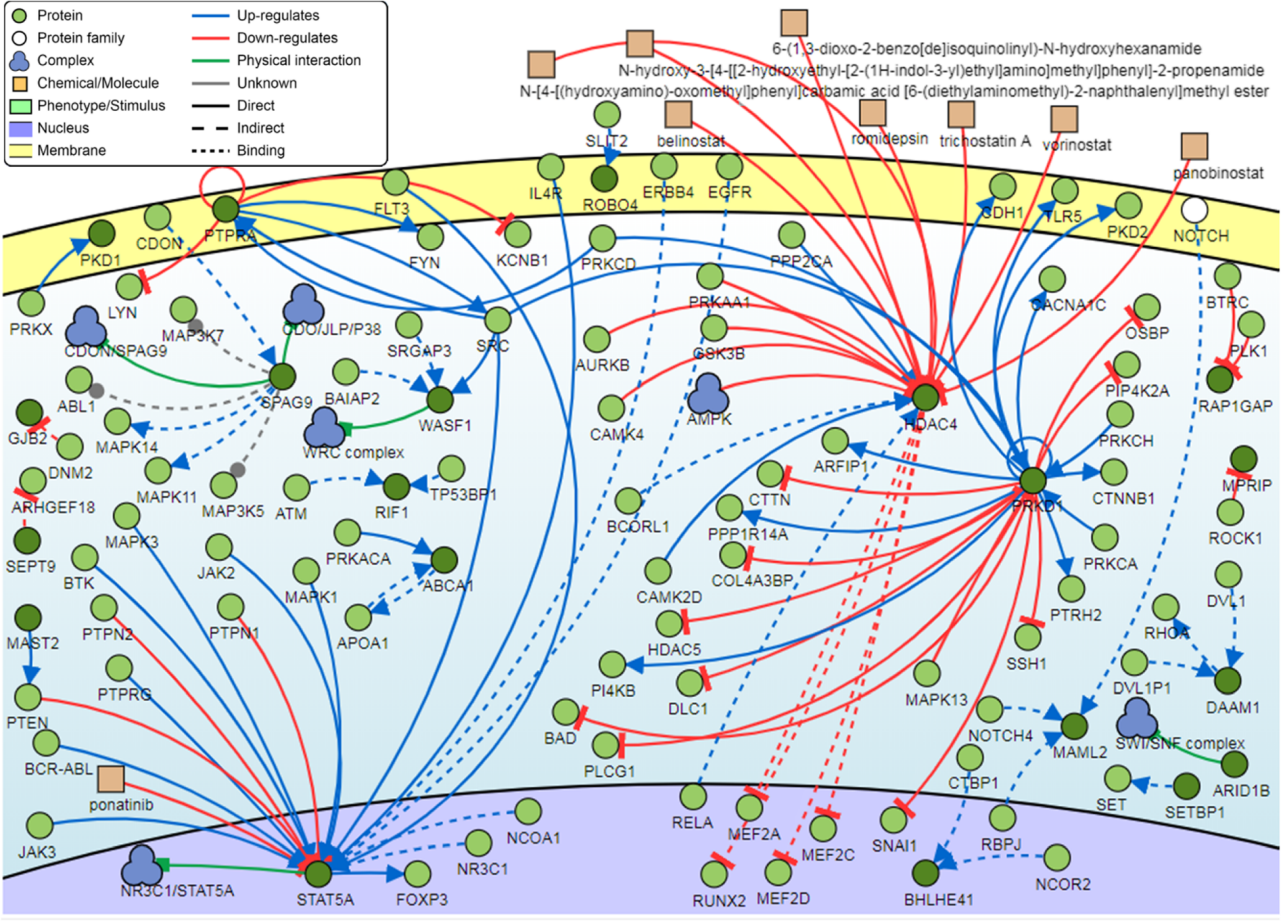
Pathway signalling network of the common gene regulators associated with the top-ranking 100 CpG sites. Three genes (*PRKD1*, *HDAC4*, and *STAT5A)* have a minimum of 20 connectivities

Analysis of the genes associated with the top 100 DM CpG sites showed that five genes were found to be common regulators with a minimum of 20 connectivities each. These genes are the *PRKD1*, *HDAC4*, and *STAT5A* genes (Fig. 8).

## Discussion

In the present study, the genome-wide methylation profile of CpG sites was demonstrated for the first time in non-genital cutaneous warts. Out of the 844,234 CpG sites that were investigated, 56,960 and 43,040 CpG sites were found to be hypomethylated and hypermethylated, respectively, in warts. The combined rank scoring method revealed the top 100 most differentially methylated CpG sites, which lay within the *C10orf26*, *FAM83H-AS1*, *ZNF644*, *LINC00702*, *GSAP*, *STAT5A*, *HDAC4*, *NCALD*, and *EXOC4* genes, among others.

cg09671951 was found to be the most hypermethylated CpG site in warts, and it is located within the *C10orf26* gene, which is also known as the outcome predictor in acute leukemia 1 (*OPAL1*) gene. The *C10orf26* gene has been associated with response to treatment in children with acute lymphoblastic leukemia, and it has also been implicated as a modulator of schizophrenia symptoms and disease progression [24–26]. The second most hypermethylated CpG site, cg27071672, lies within the *FAM83H-AS1* gene, which codes for the FAM83H antisense RNA 1 (head to head). *FAM83H-AS1* dysregulation has been associated with carcinogenesis in breast, colorectal, and lung cancer [27–29]. Two of the most hypermethylated CpG sites, cg07385604 and cg01890417, were located within the *ZNF644* gene, which encodes the zinc finger protein 644. ZNF644 is associated with transcriptional repression as a part of the G9a/GLP complex, and mutations in this gene are responsible for a monogenic form of myopia [30, 31].

cg12432168, located with the *LINC00702* gene, and cg06305962, located within the *GSAP* gene, were the fourth and fifth most hypermethylated CpG sites, respectively. The long intergenic non-protein coding RNA 702 (*LINC00702*), like other long non-coding RNAs, functions in genetic and epigenetic regulation, and its upregulation has been reported in endometrial cancer as well as malignant meningioma [32, 33]. However, the γ-secretase activating protein (*GSAP*) has mostly been reported in the context of Alzheimer’s disease pathology [34, 35]. Comparatively little is known about functions of the *LINC00702* and *GSAP* genes outside of a disease context.

In contrast, three of the most hypermethylated CpG sites (cg08246644, cg20400915, and cg08569613) were located within the signal transducer and activator of transcription 5A (*STAT5A*) gene, the latter of which has been extensively studied and elucidated. *STAT5A* has an essential function in lactogenic and mammopoietic signaling and development in adults, and its expression is upregulated by the tumor protein p53 [36, 37]. Aberrant *STAT5A* expression has been reported in a number of different cancers, including breast, colon, head and neck, and prostate cancer as well as leukemia [38–42]. Of particular interest is the association of *STAT5A* dysregulation with head and neck squamous carcinoma, which is a type of cancer that can be caused by high-risk HPV infection [43, 44]. Although low-risk HPV types lack the carcinogenic potential of their high-risk counterparts, it is intriguing that both the benign and cancerous manifestations of HPV infection exhibit aberrant *STAT5A* expression.

A further three of the most hypermethylated CpG sites (cg05171197, cg19449565, and cg17356718) were found within the histone deacetylase 4 (*HDAC4*) gene that functions in the condensation of chromatin and repression of transcription via deacetylation [45]. The survival and growth of multiple myeloma is regulated by the HDAC4-RelB-p52 complex, and the disruption of the latter blocks the growth of these cells [46]. Moreover, HDAC4 degradation by certain chemotherapeutic agents results in the apoptosis of head-and-neck cancer cells that are resistant to TRAIL, while miR-22-driven *HDAC4* repression helped to resensitize fulvestrant-resistant breast cancer cells [47, 48]. Likewise, eptoposide resistance in human A549 lung cancer cells was conferred by STAT1-HDAC4 upregulation, and *HDAC4* inhibition has been reported to induce apoptosis in non-small cell lung cancer PC-9 cells [49, 50].

HDAC4 has been previously implicated in viral replication as well as the host’s antiviral response [51]. For example, HIV-1 DNA integration is facilitated by the involvement of HDAC4 in the post-integration repair process [52]. Moreover, infection with the influenza A virus has been reported to cause airway remodeling in asthmatic individuals via the indirect dysregulation of HDAC4 [53]. HDAC4 is also a critical regulator of antiviral response, and its overexpression hinders the host immune response by suppressing type 1 interferon production [54]. Furthermore, STAT-HDAC4 signaling was reported to induce epithelial-mesenchymal transition, a malignant tumor feature that is also exhibited by keratinocytes during tissue repair [55–57]. High-risk HPV infection can similarly result in malignancy by inducing this transition in epithelial and keratinocyte cells [58–60].

With regard to functional enrichment analysis of the top-ranking 100 DM CpG sites, the most significantly enriched genes in warts were associated with SH3 domain binding, namely the Rho GTPase activating protein 31 (*ARHGAP31*), zinc finger protein 106 (*ZNF106*), synaptic Ras GTPase-activating protein 1 (*SYNGAP1*), and citron Rho-interacting serine/threonine kinase (*CIT*) genes. Despite the fact that the SH3 domain plays a role in a range of different fundamental cellular processes, not much is known about the aforementioned genes in the context of skin pathology or HPV infection [61].

In contrast, pathway analysis revealed that the Rap1 signaling pathway was the most significantly enriched term, which included the RAP1 GTPase activating protein (*RAP1GAP*), adenylyl cyclase type 9 (*ADCY9*), signal-induced proliferation-associated 1 like protein 1 (*SIPA1L1*), Rap guanine nucleotide exchange factor (GEF) 4 (*RAPGEF4*), and protein kinase D3 (*PRKD3*) genes. *RAP1GAP* downregulation via promoter hypermethylation was reported to promote the cell proliferation, survival, and migration of melanoma cells [62]. Moreover, sequence analysis of the high-risk HPV 16 E6-binding protein showed that it had the highest degree of homology with the mammalian Rap1GAP protein [63]. In addition, *PRKD3* has been previously reported to have an important role in promoting the growth and progression of invasive breast cancer [64].

Signaling network analysis of the top-ranking 100 CpG sites identified three common regulators: the protein kinase D1 (*PRKD1*), histone deacetylase 4 (*HDAC4*), and signal transducer and activator of transcription 5A (*STAT5A*) genes. The *PRKD1* gene plays an integral role in anti-differentiative and proliferative keratinocyte processes, and its aberrant expression has been suggested to have a putative tumorigenic function in the skin [65, 66]. Similarly, the *STAT5A* gene has been reported to play a major role in the keratinocyte differentiation process [67]. In the context of HPV infection, STAT5A was found to promote HPV viral replication, and STAT-5 isoforms have been indicated to contribute to the progression of HPV-associated cervical cancer [68, 69].

## Conclusions

The current study reported a number of novel CpG sites that were differentially methylated in non-genital cutaneous warts compared to normal skin. Such differences in methylation status could be responsible for the HPV-induced wart formation process. The identification of methylation status for the most differentially methylated CpG sites may prove beneficial towards the understanding of the epigenetic factors associated with non-genital cutaneous warts. One limitation of the present study is the relatively small sample size, which may result in sub-optimal statistical power for the genome-wide methylation analysis. Future research is required to validate the results on a larger scale.

## Supplementary information

**Additional file 1.Supplementary file.** Complete processed methylation data for CpG sites.

## Data Availability

The data generated over the course of the present study are available from the corresponding author upon request. However, the complete processed methylation data for the CpG sites is available as a Supplementary file.

## Abbreviations

AGRF: Australian Genome Research Facility
BP: Biological process
CC: Cellular component
CpG: 5′-C-phosphate-G-3′
DAVID: Database for Annotation, Visualization, and Integrated Discovery
DM: Differentially methylated
DNA: Deoxyribonucleic acid
GO: Gene ontology
HPV: Human papillomavirus
IRB: Institutional Review Board
JUST: Jordan University of Science and Technology
MDS: Multi-dimensional scaling
MF: Molecular function
NS: Normal skin
PCA: Principal component analysis
SIGNOR: Signaling Network Open Resource 2.0
W: Wart
